# Corneal enantiomorphism detected by Scheimpflug imaging in patients from a primary healthcare practice in Northern Poland

**DOI:** 10.1177/11206721261444811

**Published:** 2026-04-25

**Authors:** Piotr Kanclerz, Julia Tubis, Xiaogang Wang, Jorge Alio

**Affiliations:** 1 Helsinki Retina Research Group, 3835 University of Helsinki, Helsinki, Finland; 2 Department of Ophthalmology, Hygeia Clinic, Gdańsk, Poland; 3 Department of Cataract, 12441 Shanxi Eye Hospital Affiliated to Shanxi Medical University, Taiyuan, China; 4 Vissum Corporation, Alicante, Spain

**Keywords:** Anisometropia, central corneal thickness, corneal asymmetry, corneal enantiomorphism, keratometry

## Abstract

**Purpose:**

Many studies have focused on the presence of corneal enantiomorphism in patients with keratoconus, and single reports have presented its occurrence among healthy individuals. This study aimed to evaluate the magnitude of enantiomorphism in a Polish general practice population representing different age groups.

**Methods:**

This cross–sectional study involved patients registered with a general practitioner. Corneal measurements were conducted using a rotating Scheimpflug corneal tomography system, and the symmetry between stereometric parameters of the cornea was analyzed.

**Results:**

The study included 590 patients (241 men and 349 women) of Polish nationality with a mean age of 53.35 ± 16.70 years. The mean keratometry values were 43.26 ± 1.40 D for the right eye and 43.27 ± 1.36 for the left eye (mean absolute interocular difference 0.01 ± 0.37 D). A difference in mean keratometry values greater than or equal to 0.75 D was noted in 29 patients (4.9%), with a difference of greater than 1 D in 10 patients (1.7%). Corneal astigmatism equal to or greater than 0.75 D was noted in 396 (67.0%) of patients, while 225 patients (38.1%) had astigmatism in both eyes. Isorule astigmatism was noted in 88.4% (199/225) patients having astigmatism in both eyes. The absolute interocular difference in central corneal thickness was 7.74 ± 6.26 µm (*p* < 0.01), and correlated with the level of astigmatism (*p* = 0.02), but not with age or mean keratometry.

**Conclusion:**

The degree of corneal enantiomorphism was high in our study population, with interocular differences in keratometric power being relatively small. This study provides reference values for interocular corneal symmetry, contributing to the understanding of enantiomorphic patterns.

## Introduction

Anatomical asymmetry occurs in every person. It is natural and should not be a cause of concern in most cases. Most paired organs exhibit some degree of asymmetry, including differences in the positioning of the kidneys and the number of lobes in the lungs. The term enantiomorphism refers to chemical molecules whose structures are mirror images of each other and are non-superimposable. In ophthalmology, enantiomorphism has been extensively studied in the context of the right and left cornea.^1^ Research comparing the degree of interocular symmetry has been focused primarily on the axis distribution and less on refractive cylinder power.^2,3^ However, evidence suggests that interocular symmetry exists not just for astigmatism but also for additional corneal-related parameters including pachymetry (corneal thickness), best-fit sphere, and other relevant factors with potential applications in clinical settings.^4^

Enantiomorphism can also play a critical role in cataract and refractive surgery. In cataract surgery, symmetry is preserved to allow for the most favorable surgical results. Increased interocular biometric differences correlate with inaccurate refractive outcomes following phacoemulsification and can lead to reduced binocular vision performance.^5^ In corneal refractive surgery, correction of the refractive error when a significant difference between the eyes is present, can lead to problems in binocular vision, difficulties in adapting to changes, and impaired stereoscopic vision.^6^ Thus, corneal enantiomorphism offers a framework for assessing bilateral corneal health, enhancing diagnostic sensitivity, optimizing refractive planning, and guiding postoperative evaluation. Moreover, its integration into clinical practice could support more precise, personalized ophthalmic care.

Many studies have focused on the occurrence of enantiomorphism in keratoconus, and the loss of corneal enantiomorphism is considered as a hallmark of keratoconus.^7^ However, single case reports have analyzed corneal enantiomorphism among healthy individuals.^8,9^ This study aimed to evaluate the degree of enantiomorphism in a Polish general practice population representing different age groups and identify associated risk factors.

## Methods

### Study design

This study was a prospective, cross-sectional analysis conducted consecutively on patients registered with a general practitioner (GP). The inclusion criteria required participants to have no prior history of corneal surgery or diagnosed ocular diseases, particularly keratoconus. Only individuals without significant ophthalmic conditions that could influence corneal parameters were selected to ensure a homogeneous study group for analysis.

### Patient acquisition

In Poland, healthcare is constitutionally guaranteed, with medical activities overseen by the Polish Ministry of Health. The National Health Fund administers public healthcare, requiring each patient to register with a GP, who manages their medical care and provides specialist referrals when necessary. A single GP typically serves up to 2,500–2,800 patients and while geographical proximity is not a primary factor in choosing a GP, patients generally prefer one located in their residential area or accessible via public transport or car.

Participants were recruited from patients registered with a single GP at the Hygeia Clinic's Elbląg branch in Poland. Eligible individuals, aged 10–80 years and of Polish ethnicity, were invited to participate via phone calls offering free examinations. Patients visiting the clinic for GP consultations were also invited to enroll in the study. The study adhered to the principles of the Declaration of Helsinki, and written consent was obtained from all participants or their guardians, as required by Polish law. Prior to the examination, participants were asked if they had undergone laser vision correction. The study protocol was approved by the local bioethics committee (Komisja Bioetyczna przy Izbie Lekarskiej w Gdańsku, approval no. KB-13/20).

### Examination

Due to the SARS-CoV-2 pandemic, both patients and examiners wore face masks, and interpersonal contact was minimized. Upon arrival, participants received an explanation of the study's purpose and signed informed consent forms.

**Figure 1.**
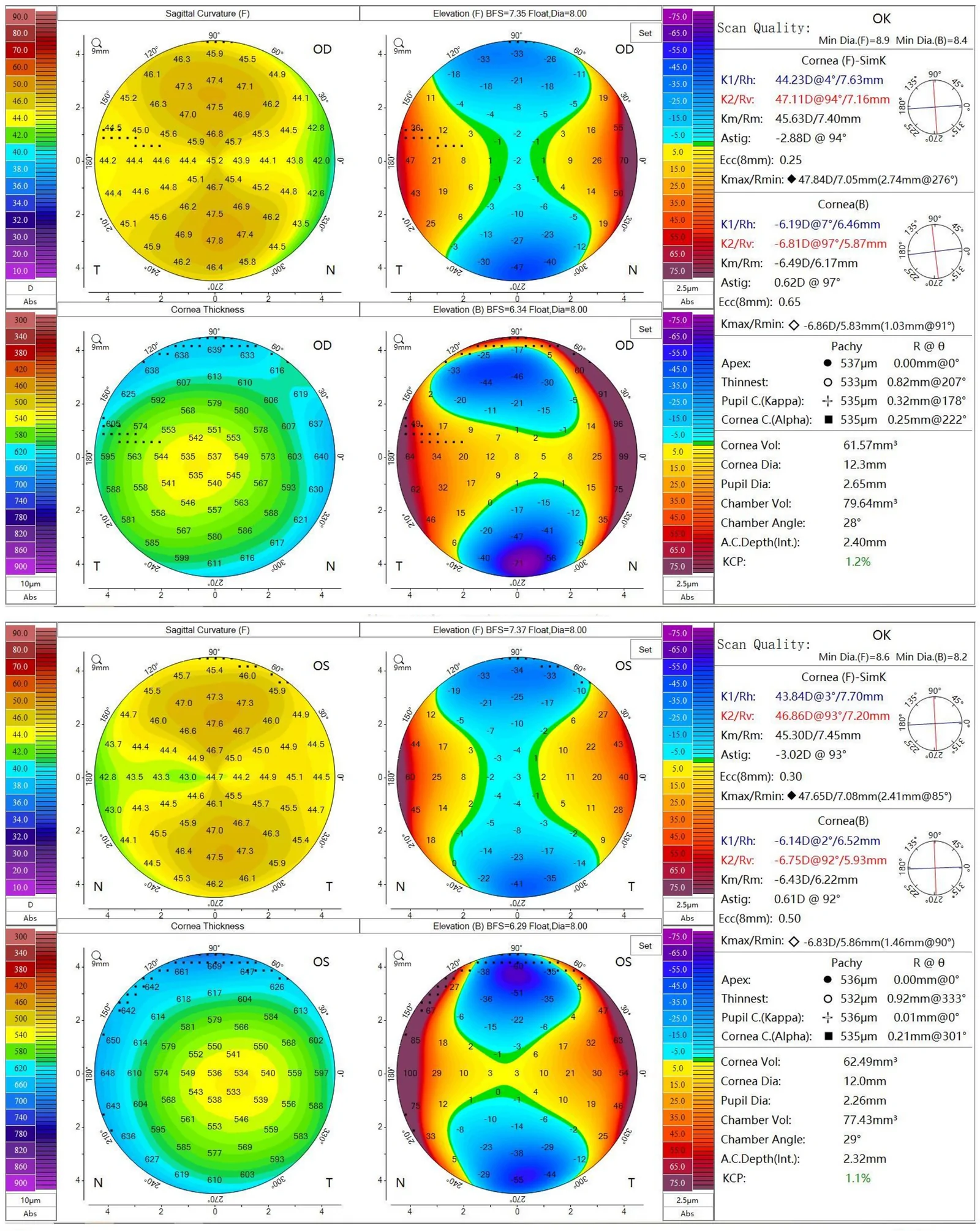
Scansys images of a 53 year-old female patient with isorule with-the-rule astigmatism. The upper four images present scans of the right eye, while the lower four images of the left eye.

Corneal images were captured using the Scansys Anterior Segment Analyzer TA 517 (MediWorks Precision Instruments, Shanghai, China), a corneal tomographer equipped with an infrared and Scheimpflug camera.^10^ This device, which has Conformité Européenne (CE) approval, collects 107,520 data points and acquires 28 images in a one-second scan. It generates cross-sectional images and three-dimensional reconstructions of the cornea, providing topographic maps of corneal curvature, thickness, and anterior/posterior surface elevation (Figure 1). This study was not designed to determine the repeatability and reproducibility of the Scansys device, however, a previous study has shown that the results obtained with Scansys could be considered interchangeable with Pentacam HR (Oculus Optikgeräte GmbH, Wetzlar, Germany) in several parameters.^11^

Examinations were conducted in a dimly lit room between 9 AM and 6 PM to accommodate participants during clinic hours. Patients using contact lenses were requested to avoid wearing them for at least 7 days prior to the examination. Each eye was imaged three times in a randomized order by experienced examiners (K.P. and J.Ś.). All scans were recorded, but only the highest-quality image was analyzed. A scan was deemed acceptable if it covered an adequate area, captured no eye movement, and allowed the device to generate accurate stereometric corneal models. Image quality was automatically verified by the software, with an “OK” indicator confirming proper acquisition (image centration, image clear of any eyelid interference, signal strength).^12^ No additional scans were performed if image quality was insufficient.

### Definitions

In our previous study, the prevalence of keratoconus in the same clinic population was assessed.^13^ Participants were asked whether they had undergone corneal refractive surgery. Those with a history of laser vision correction, ocular trauma, diagnosis of keratoconus or suspected keratoconus (within our previous on the same cohort)^13^ were excluded. Only individuals with high-quality images in both eyes were included in the analysis.

Corneal anisometropia was defined as the difference between the mean keratometry values of the left and right eyes; the prevalence of corneal anisometropia was evaluated with cutoff values equal to or greater than 0.75 D, greater than 1.0 D, and greater than 2.0 D. Astigmatism was defined as cylinder power equal to or greater than 0.75 D; however, we also analyzed the prevalence rates for astigmatism equal to or greater than 0.50, 1.00, 2.00 and 3.00 D. As measurements on two eyes of one subject are usually related and not independent, outcomes for both the right eye, left eye and r person-level (prevalence in right or left eye) outcomes were presented.^14,15^ The classification of astigmatism was based on the axis of the flat corneal meridian: (i) between 0° and 30° or between 150° and 180° - with-the-rule astigmatism, (ii) between 60° and 120° - against-the-rule astigmatism, (iii) any other meridian - oblique astigmatism. Cylindrical corneal anisometropia was defined as a cylinder power difference of ≥1 D between the right and left eyes.^16^ Astigmatism was expressed in both polar and vector forms, with vector components J0 and J45 calculated per Thibos et al.^17^ Cases of bilateral astigmatism were classified into two groups: (i) isorule astigmatism: both eyes had the same orientation (with-the-rule, against-the-rule or oblique), (ii) anisorule astigmatism: fellow eyes had different orientations (e.g., one with-the-rule, the other against-the-rule).^9^ In isorule astigmatism, the fellow eyes of an individual had similar orientations (both eyes with-the-rule, against-the-rule or oblique astigmatism).^9^

### Primary outcome measures

The primary outcomes included key corneal parameters: mean keratometry, steep and flat keratometry axis, astigmatism magnitude, and central corneal thickness (CCT). These parameters were analyzed for the right eye and compared between the right and left eyes to assess enantiomorphism levels.

### Statistical analysis

Statistical analysis was performed using MedCalc software (MedCalc Software bvba, Ostend, Belgium) and GNU PSPP 2.0.1 (Free Software Foundation, Boston, Massachusetts, USA). Descriptive statistics summarized demographic data and prevalence rates. Correlations were analyzed using Pearson coefficients, intraclass correlation coefficient (ICC), Bland-Altman plots, and paired t-tests, as all parameters followed a normal distribution (Kolmogorov–Smirnov test). Pearson correlation coefficient interpretations: (i) **0–0.3:** weak positive correlation, (ii) **0.3–0.7:** moderate positive correlation, (iii) **0.7–1.0:** strong positive correlation.^18^ A p-value less than 0.05 was considered statistically significant.

## Results

**Figure 2.**
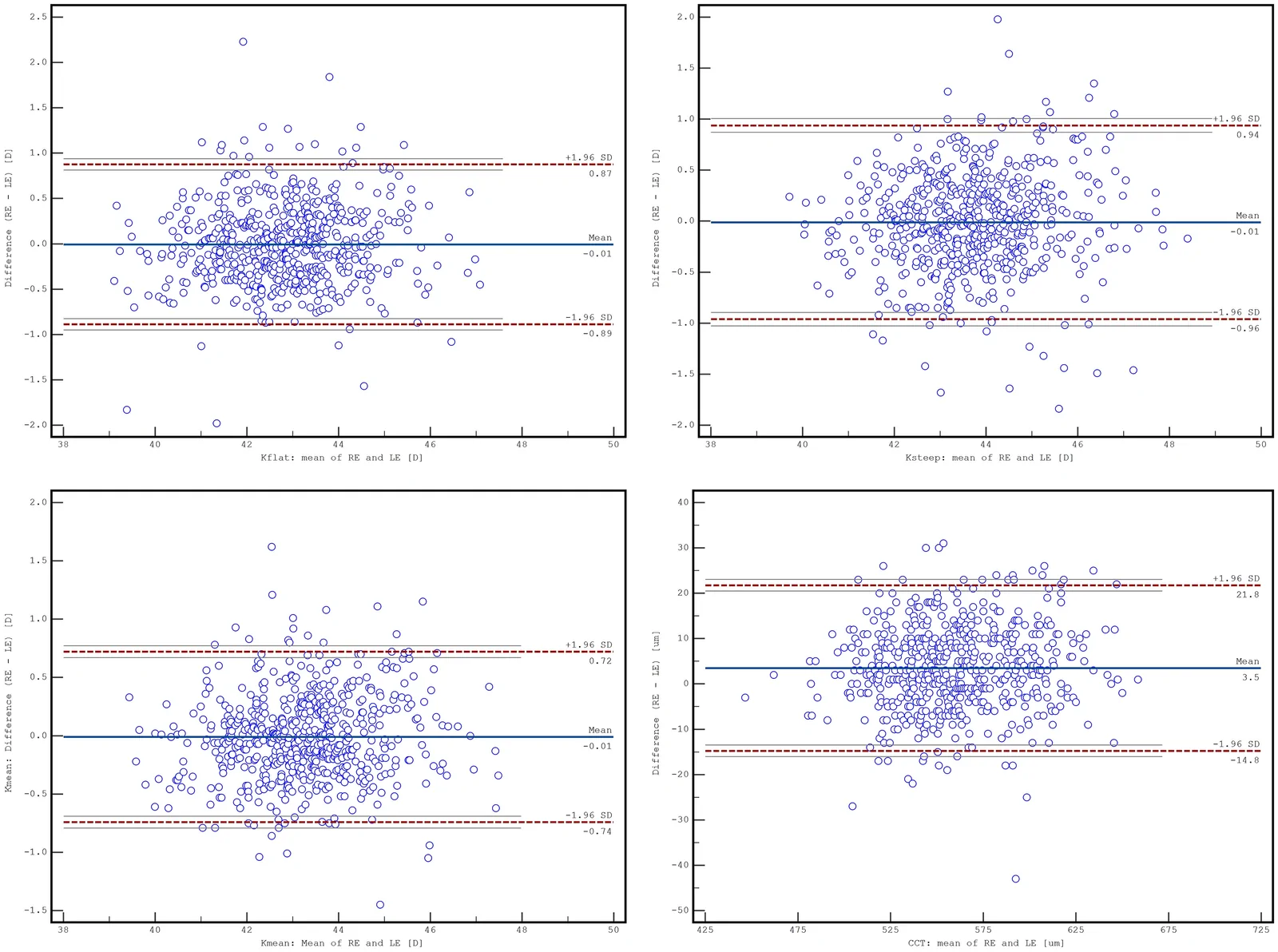
The Bland-Altman plots demonstrating the agreement between the right eye (RE) and left eye (LE) for flat (a) and steep (b) keratometry, mean keratometry (c), and central corneal thickness (CCT) (d).

The study included 590 patients (241 men and 349 women) of Polish nationality, aged between 10 and 80 years. Their mean age was 53.35 ± 16.70 years. The mean keratometry values were 43.26 ± 1.40 D for the right eye and 43.27 ± 1.36 for the left eye (*p* = 0.50; Table 1). The mean keratometric difference between the right and left eye was 0.01 ± 0.37 D. However, when absolute values for the difference were taken into consideration, the mean difference was 0.29 ± 0.24 D. The steep keratometry was 43.70 ± 1.46 D for the right eye and 43.71 ± 1.42 D for the left eye (*p* = 0.55), with absolute values of the difference 0.37 ± 0.31 D. The mean value of flat keratometry for the right eye was 42.80 ± 1.40 D and for the left eye 42.81 ± 1.37 D (*p* = 0.72), with absolute values of the difference 0.34 ± 0.29 D. The Bland-Altman plots demonstrating the agreement between the right eye and left eye for keratometric values and CCT has been presented in Figure 2. There was a strong positive interocular correlation for Kmean readings (r = 0.96; ICC = 0.96 (95% confidence interval [CI]: 0.95 to 0.97)), as well as for Kflat (r = 0.95; ICC = 0.95; 95% CI: 0.94 to 0.96), and for Ksteep readings (r = 0.94; ICC = 0.94; 95% CI: 0.93 to 0.95; Supplementary Table 1). Univariate analysis has shown that Kmean readings were associated with sex (*p* < 0.01), age (*p* < 0.01; r = 0.21) and CCT (*p* = 0.01; r = −0.11). Similarly, multiple logistic regression analysis showed that Kmean was associated with sex (*p* < 0.001), age (*p* < 0.01) and CCT (*p* = 0.02).

**Table 1.** The differences and level of correlation between corneal parameters for the right and left eye (n = 590).

Parameter	Right eye		Left eye		P value	Right eye minus left eye		Absolute value of right eye minus left eye		Correlation coefficien: *r* (p)	ICC (95% CI)
	Mean ± SD	95% CI	Mean ± SD	95% CI		Mean ± SD	95% CI	Mean ± SD	95% CI		
Kmean [D]	43.26 ± 1.40	43.14 to 43.37	43.27 ± 1.36	43.15 to 43.37	0.50	0.01 ± 0.37	−0.02 to 0.04	0.29 ± 0.24	0.27 to 0.31	0.96 (<0.01)	0.96 (0.95 to 0.97)
Kflat [D]	42.80 ± 1.40	42.69 to 42.91	42.81 ± 1.37	42.70 to 42.92	0.72	0.01 ± 0.45	−0.03 to 0.04	0.34 ± 0.29	0.32 to 0.37	0.95 (<0.01)	0.95 (0.94 to 0.96)
Ksteep [D]	43.70 ± 1.46	43.58 to 43.82	43.71 ± 1.42	43.60 to 43.83	0.55	0.01 ± 0.48	−0.03 to 0.05	0.37 ± 0.31	0.34 to 0.40	0.94 (<0.01)	0.94 (0.93 to 0.95)
CCT [μm]	561.70 ± 34.42	558.91 to 564.48	558.20 ± 33.63	555.48 to 560.92	<0.01	−3.50 ± 9.32	−4.25 to −2.74	7.74 ± 6.26	7.23 to 8.24	0.96 (<0.01)	0.96 (0.95 to 0.97)
Corneal astigmatism [D]	0.91 ± 0.64	0.85 to 0.96	0.91 ± 0.66	0.85 to 0.96	0.97	0.00 ± 0.56	−0.04 to 0.05	0.40 ± 0.39	0.37 to 0.43	0.63 (<0.01)	0.62 (0.56 to 0.68)
Axis	99.16 ± 68.11	93.65 to 104.67	90.45 ± 69.27	84.85 to 96.05	0.04	8.71 ± 102.35	0.45 to 16.97	79.98 ± 1.67	74.78 to 85.19	−0.11 (0.01)	−0.11 (−0.19 to −0.03)
J0	−0.28 ± 0.43	−0.32 to −0.25	−0.31 ± 0.45	−0.34 to −0.27	0.16	0.03 ± 0.40	0.00 to 0.60	0.24 ± 0.33	0.21 to 0.27	0.58 (<0.01)	0.58 (0.52 to 0.63)
J45	0.05 ± 0.26	0.03 to 0.07	−0.02 ± 0.28	−0.04 to 0.00	<0.01	0.02–0.49	−0.02 to 0.06	0.32 ± 0.38	0.29 to 0.35	−0.25 (<0.01)	−0.24 (−0.31 to −0.16)

CCT: central corneal thickness; CI: confidence interval; ICC: intraclass correlation coefficient; J0 and J45: vector values of astigmatism; Kflat: flat keratometry; Kmean: mean keratometry; Ksteep: steep keratometry

**Figure 3.**
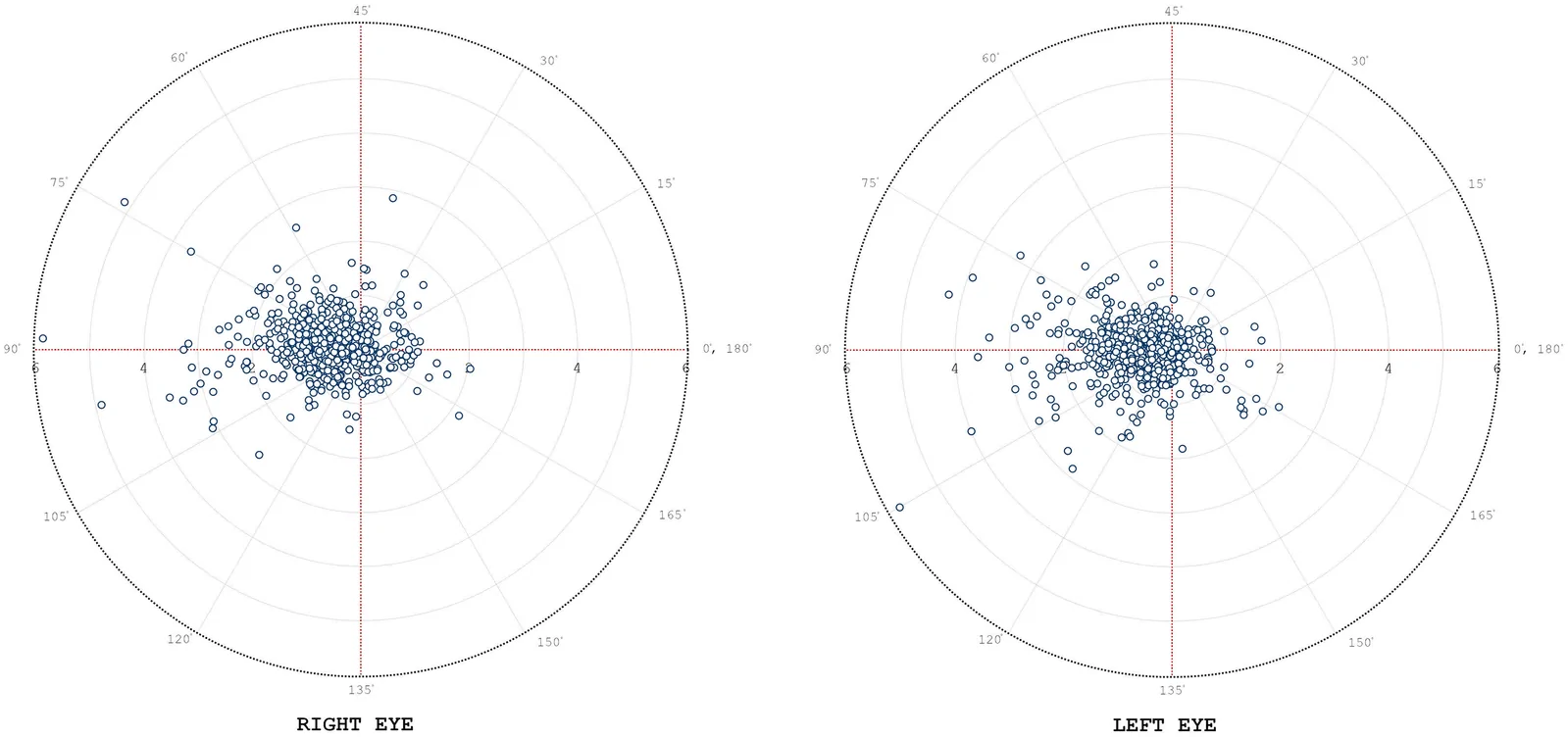
Double-angle plot of corneal astigmatism in the right and left eyes. Most eyes have with-the-rule astigmatism (axis at 90°).

Corneal astigmatism of equal to or greater than 0.75 D was noted in 396 patients (67.0%), while 225 patients (38.1%) had astigmatism in both eyes. A double-angle plot of corneal astigmatism for the right and left eye has been presented in Figure 3. The prevalence of astigmatism equal to or greater than 1D, greater than 1D, equal to or greater than 2D, and equal to or greater than 3D in the right eye was 47.2% (279/590 patients), 46.7% (276/590 patients), 10.7% (63/590 patients), and 3.2% (19/590 patients), respectively (Table 2). Corneal cylindrical anisometropia was 0.01 ± 0.05 D. Among all patients, 36 (6.1%), 7 (1.2%), and 3 (0.05%) had corneal cylindrical anisometropia in corneal power of equal to or greater than 1 D, 2 D, and 3 D, respectively. Isorule astigmatism was noted in 199 out of 225 patients having astigmatism in both eyes (88.4%), and 26 eyes had anisorule astigmatism (11.6%; Table 3).

**Table 2.** Prevalence of astigmatism by cylinder power in the study cohort.

Threshold	Person level-outcome (in right or left eye)	Prevalence in right eyes (n = 590)	Prevalence in left eyes (n = 590)	Astigmatism in the right and left eye
0.50 D or more	85.8% (507)	72.9% (431)	69.7% (412)	56.9% (336)
0.75 D or more	67.0% (396)	53.7% (317)	51.4% (304)	38.2% (226)
1 D or more	47.2% (279)	34.2% (202)	34.4% (203)	21.5% (127)
More than 1.00 D	46.7% (276)	33.6% (198)	34.0% (201)	21.0% (124)
2.00 D or more	10.7% (63)	6.8% (40)	7.1% (42)	3.6% (21)
More than 2.00 D	10.7% (63)	6.8% (40)	7.1% (42)	3.6% (21)
3.00 D or more	3.2% (19)	1.9% (11)	1.4% (8)	0.3% (2)
More Than 3.00 D	3.2% (19)	1.9% (11)	1.4% (8)	0.3% (2)

**Table 3.** Agreement in astigmatism subtypes between the right and left eye (total n = 590).

	Right eye				
Left eye		No	With-the-rule	Against-the-rule	Oblique
	No	194 (32.8%)	63 (10.7%)	17 (2.9%)	12 (2.0%)
	With-the-rule	61 (10.3%)	186 (31.5%)	2 (0.3%)	9 (1.5%)
	Against-the-rule	5 (0.01%)	3 (0.5%)	11 (1.9%)	2 (0.3%)
	Oblique	12 (2.0%)	7 (1.2%)	3 (0.5%)	2 (0.3%)

An interocular difference in mean keratometry values greater than or equal to 0.75 D was noted in 29 patients (4.9%), with a difference of greater than 1 D in 10 patients (1.7%). No patients demonstrated a difference of greater than 2 D. Univariate analysis has shown that corneal anisometropia was associated with the level of astigmatism (odds ratio [OR]: 3.06; 95% CI: 1.90–4.92; *p* < 0.01), but not age (OR: 1.01; 95% CI: [0.93–1.05]; *p* = 0.369), male sex (OR: 2.34; 95% CI 0.65–8.59; *p* = 0.583), mean keratometry (OR: 1.52; 95% CI: 1.04–2.24; *p* = 0.149), or pachymetry at the corneal apex (OR: 1.00; 95% CI: 0.99–1.02; *p* = 0.708). Multiple logistic regression analysis showed that corneal astigmatism was associated with corneal anisometropia (*p* < 0.01), but sex (*p* = 0.818), age (*p* = 0.105) and pachymetry at the corneal apex (*p* = 0.48) were not.

The CCT was 561.70 ± 34.42 µm in the right eye, and 558.20 ± 33.6 µm in the left eye. Univariate analysis has shown that CCT was associated with age (*p* = 0.02; r = −0.09), and the mean keratometry value (*p* = 0.22; r = −0.11), but not with sex (*p* = 0.55) or the level of astigmatism (*p* = 0.71). Regression analysis showed that the CCT was associated with age (*p* = 0.02) and mean keratometry (*p* = 0.01) but not with the level of astigmatism (*p* = 0.39) or sex (*p* = 0.96). The mean absolute interocular difference in CCT was 7.74 ± 6.26 µm (*p* < 0.01). The mean absolute difference in CCT has shown association with the level of astigmatism (*p* = 0.02), but not with age (*p* = 0.07) or mean keratometry (*p* = 0.07).

## Discussion

The difference in keratometric values between eyes was relatively low and showed interocular symmetry (r = 0.96 for mean keratometry). The concept of corneal enantiomorphism has been most extensively studied in patients with keratoconus. As previously demonstrated by Cavas-Martínez et al.,^19^ the loss of enantiomorphism is a key indicator of early ectatic changes, making corneal symmetry a crucial factor in our analysis. Zadnik et al. noted that in patients with keratoconus, the average anisometropia was 3.0 D and the mean corneal curvature differed by 3.6–4.4 D between eyes.^20^ Eppig et al. demonstrated interocular differences in patients with keratoconus in various parameters, including corneal power, corneal thickness, and topographic and biomechanical indices.^21^ In that study the asymmetry in Kmean was significantly greater in patients with keratoconus than in healthy controls (3.8 ± 4.0 D vs. 0.22 ± 0.17 D, respectively). CCT asymmetry was also greater among patients with keratoconus than among healthy controls (34 ± 30 vs. 6 ± 5 μm). Naderan et al. showed that particularly maximum keratometry, Kflat and anterior corneal elevation might be most accurate in identifying keratoconus.^22^ In our study, interocular differences in mean keratometry greater than 0.31 D could be considered outside the 95% CI, and could require further clinical attention in ocular surgery. Similarly an interocular difference of at least 0.37 D in Kflat, 0.40 D in Ksteep and at least 8.25 μm in CCT could require additional evaluation. However, Shen et al. suggested cut-offs of 0.75 D for the Kmean, 0.67 D for the keratometric standard deviation, 2.9 μm for standard deviation of the front corneal elevation, and 14.6 μm for maximum front corneal elevation as parameters with the greatest sensitivities (95.7%, 95.0%, 96.9%, and 95.0%, respectively) and specificities (96.0%, 97.7%, 94.8%, and 95.4%, respectively) for detecting keratoconus.^23^ These data reinforce the importance of carefully evaluating interocular differences in corneal parameters, both for identifying early ectatic changes and for optimizing surgical planning.

In our study, no patients demonstrated an interocular difference in mean keratometry of at least 2 D, and only 1.7% had an interocular difference of greater than 1 D. Pearson's correlation coefficient between the of Kflat, Ksteep and Kmean between the right and left eye was very strong (r = 0.95; r = 0.94 and r = 0.96, respectively). These findings are consistent with Xu et al., who reported similar degrees of enantiomorphism in terms of corneal curvature in a Chinese myopic population (r = 0.97, r = 0.96 and r = 0.98, respectively).^24^ However, our study demonstrated greater enantiomorphism in terms of the degree of corneal astigmatism (0.91 ± 0.64 for the right eye, and 0.91 ± 0.66 for the left eye; r = 0.97) than Xu et al. (1.09 ± 0.61 for the right eye, and 1.07 ± 1.10 for the left eye; r = 0.78).^24^ With-the-rule astigmatism was the most common in the evaluated cohort, consistent with previous studies.^25,26^ Our study also showed that among patients with corneal astigmatism in both eyes, 88.4% had isorule astigmatism, whereas 11.6% had anisorule astigmatism. The prevalence of astigmatism equal to or greater than 0.5 D in both eyes was 56.9%, while in the study by Hashemi et al. 18.42% of patients has astigmatism in both eyes.^9^ The differences in the prevalence of astigmatism between studies may reflect differences in demographic compositions of the study populations, including the age distribution of the population, disparity in healthcare access, and ethnicity. Moreover, environmental risk factors and lifestyle habits prevalent in each country might influence the occurrence and distribution of different types of astigmatism.

Recent studies have shown that CCT measurements are usually highly symmetric between eyes, with almost 7% of patients manifesting significant interocular CCT asymmetry.^27–29^ A single study has shown that CCT negatively correlated with age (r = −0.263, *p* < 0.05), decreasing from 560.3 ± 24.3 μm in individuals aged 30–39 years to 530.9 ± 15.2 μm in those aged 80 years and older.^30^ Our study also showed a significant but weaker negative correlation between CCT and age (r = −0.09; *p* = 0.02). Significant attention has been given to CCT disparities between healthy and keratoconic eyes. Shen et al. found a higher mean CCT in patients with keratoconus (469.5 ± 53.7 μm) compared to controls (546.6 ± 33.4 μm).^23^ The mean interocular difference in CCT was also greater among patients with keratoconus than among healthy controls (21.5 ± 13.6 µm vs. 11.8 ± 4.2 µm).^23^ In our study, the mean absolute interocular difference in CCT was 7.74 ± 6.26 µm (*p* < 0.01). Asymmetric CCT has also been associated with asymmetric primary open-angle glaucoma.^31^ Iester et al. demonstrated that 18% of patients with glaucoma exhibited interocular differences in CCT of >20 μm, but found no clear link between variations in CCT variations and glaucoma-induced damage.^32^ Similarly, Sullivan-Mae et al. found that in patients with glaucoma and asymmetric corneal thickness, the eye with the thinner cornea was more likely to exhibit worse visual field loss, especially when the thickness difference exceeded 15 µm.^31^ Although the mechanism has not been fully elucidated, it has been suggested that thinner corneas may indicate greater structural susceptibility to glaucomatous damage.^31^

This study has several limitations that should be acknowledged. Firstly, its single-center design may have introduced recruitment bias, as all participants were drawn from one local primary care population. Therefore, our study population may not be representative of the broader population of Northern Poland or other regions of Poland due to potential regional differences in demographic structure, referral patterns, socioeconomic statuses, health-seeking behaviors, environmental exposures, and access to eye care. Secondly, the patients who agreed to participate in this study may differ systematically from those who declined to participate, such as being more health-conscious, potentially further skewing estimates. Thirdly, normality was defined solely by having non-keratoconic tomography results, not based on a comprehensive eye examination (e.g., slit lamp, refraction, visual acuity, and corneal biomechanics). Therefore, our findings should be interpreted with caution and ideally be confirmed through multicenter studies spanning diverse communities.

In conclusion, the degree of corneal enantiomorphism was high in our study population, with interocular differences in keratometric power being relatively small. This study provides reference values for interocular corneal symmetry in a general practice population, contributing to the understanding of enantiomorphic patterns.

## Supplemental Material

sj-doc-1-ejo-10.1177_11206721261444811 - Supplemental material for Corneal enantiomorphism detected by Scheimpflug imaging in patients from a primary healthcare practice in Northern Poland Supplemental material, sj-doc-1-ejo-10.1177_11206721261444811 for Corneal enantiomorphism detected by Scheimpflug imaging in patients from a primary healthcare practice in Northern Poland by Piotr Kanclerz, Julia Tubis, Xiaogang Wang and Jorge Alio in European Journal of Ophthalmology
