# Supplementary material for: Corneal enantiomorphism detected by Scheimpflug imaging in patients from a primary healthcare practice in Northern Poland
Source: Eur J Ophthalmol. 2026 Apr 25;36(5):1323–31. doi: 10.1177/11206721261444811 (PMC13490678; doi:10.1177/11206721261444811)
Supplement: Supplementary file 1 [file sj-doc-1-ejo-10.1177_11206721261444811.doc]

**Supplementary Table 1. Distribution of flat, steep and mean keratometry, within the study population (n=590)**

|  | **Kflat [D]** | **Ksteep [D]** | **Kmean [D]** | **CCT [μm]** |
| --- | --- | --- | --- | --- |
| **Laterality** |  |  |  |  |
| right eye | 42.80 ± 1.40 | 43.70 ± 1.46 | 43.26 ± 1.40 | 561.70 ± 34.42 |
| left eye | 42.81 ± 1.37 | 43.71 ± 1.42 | 43.27 ± 1.36 | 558.20 ± 33.63 |
|  | p = 0.72; r = 0.95 ICC = 0.95 (0.94 to 0.96) | p = 0.73; r = 0.94 ICC = 0.94 (0.93 to 0.95) | p = 0.50; r = 0.96  ICC = 0.96 (0.95 to 0.97) | p < 0.01; r = 0.96  ICC = 0.96 (0.95 to 0.97) |
| **Sex** |  |  |  |  |
| male | 42.46 ± 1.42 | 43.29 ± 1.43 | 42.87 ± 1.40 | 562.02 ± 33.85 |
| female | 43.06 ± 1.38 | 44.00 ± 1.42 | 43.56 ± 1.34 | 561.93 ± 34.55 |
|  | p < 0.01 | p < 0.01 | p < 0.01 | p = 0.98 |
| **Age [years]** |  |  |  |  |
| 30 or less | 42.21 ± 1.36 | 43.20 ±1.53 | 42.68 ± 1.39 | 572.35 ± 36.40 |
| 30-40 | 42.54 ± 1.45 | 43.54 ± 1.54 | 43.04 ± 1.47 | 559.85 ± 28.69 |
| 40-50 | 42.60 ± 1.64 | 43.64 ± 1.64 | 43.13 ± 1.54 | 561.16 ± 37.67 |
| 50-60 | 42.85 ± 1.32 | 43.70 ± 1.37 | 43.27 ± 1.30 | 562.10 ± 33.69 |
| 60-70 | 43.02 ± 1.31 | 43.83 ± 1.34 | 43.42 ± 1.30 | 559.51 ± 32.94 |
| 70 or more | 43.41 ± 1.30 | 44.22 ± 1.39 | 43.82 ± 1.31 | 561.46 ± 32.60 |
|  | p < 0.01; r = 0.24 | p < 0.01; r = 0.17 | p < 0.01; r = 0.21 | p = 0.02; r = -0.10 |
